# A Novel Sampling Method to Measure Socioeconomic Drivers of *Aedes albopictus* Distribution in Mecklenburg County, North Carolina

**DOI:** 10.3390/ijerph15102179

**Published:** 2018-10-05

**Authors:** Ari Whiteman, Eric Delmelle, Tyler Rapp, Shi Chen, Gang Chen, Michael Dulin

**Affiliations:** 1 Department of Geography and Earth Sciences and Center for Applied Geographic Information Science, University of North Carolina at Charlotte, 2901 University City Blvd, Charlotte, NC 28223, USA; delmelle@gmail.com (E.D.); gang.chen@uncc.edu (G.C.); 2 Academy for Population Health Innovation, 9201 University City Blvd, Charlotte, NC, 28223, USA; trapp1@uncc.edu (T.R.); mdulin3@uncc.edu (M.D.); 3 Department of Public Health Sciences, University of North Carolina at Charlotte, 2901 University City Blvd, Charlotte, NC 28223, USA; schen56@uncc.edu

**Keywords:** vector-borne disease, health disparities, optimization, social determinants of health

## Abstract

Climate change, urbanization, and globalization have facilitated the spread of *Aedes* mosquitoes into regions that were previously unsuitable, causing an increased threat of arbovirus transmission on a global scale. While numerous studies have addressed the urban ecology of *Ae. albopictus*, few have accounted for socioeconomic factors that affect their range in urban regions. Here we introduce an original sampling design for *Ae. albopictus*, that uses a spatial optimization process to identify urban collection sites based on both geographic parameters as well as the gradient of socioeconomic variables present in Mecklenburg County, North Carolina, encompassing the city of Charlotte, a rapidly growing urban environment. We collected 3645 specimens of *Ae. albopictus* (87% of total samples) across 12 weeks at the 90 optimized site locations and modelled the relationships between the abundance of gravid *Ae. albopictus* and a variety of neighborhood socioeconomic attributes as well as land cover characteristics. Our results demonstrate that the abundance of gravid *Ae. albopictus* is inversely related to the socioeconomic status of the neighborhood and directly related to both landscape heterogeneity as well as proportions of particular resident races/ethnicities. We present our results alongside a description of our novel sampling scheme and its usefulness as an approach to urban vector epidemiology. Additionally, we supply recommendations for future investigations into the socioeconomic determinants of vector-borne disease risk.

## 1. Introduction

As a result of the proliferation of container-breeding *Aedes* mosquitoes across much of the world’s urban regions, rates of arbovirus infections have increased globally over the last several decades [1,2,3]. Subsequently, research has been initiated to better understand the ecology of the urban *Aedes* mosquitoes that act as vectors for the most commonly transmitted viruses in these urban regions, including: dengue virus, yellow fever virus, chikungunya virus, and Zika virus. Studies of the most abundant urban *Aedes* species, *Ae. albopictus* and *Ae. aegypti*, have investigated breeding behavior [4,5], feeding behavior [6,7], and habitat preferences [8,9]. This work has produced vital information that has advanced vector control efforts and reduced disease transmission. However, with climate change [10], globalization [11], and urbanization [12] threatening to spur further increases in arbovirus transmission rates, it is important to both evaluate prior methods of vector surveillance as well as test novel sampling designs that will improve current practices of designing mosquito surveillance systems.

There have been numerous studies of mosquito trapping techniques aimed at determining the sampling methods that most accurately account for known aspects of mosquito ecology [13,14,15,16,17,18,19,20,21,22,23]. Additionally, the accuracy of commonly used entomological indexes have also been examined [24,25,26,27]. To date, the majority of these studies focus on differences in trap types rather than arrangement of the traps in the geographic space. In most studies on *Aedes* population distribution, survey site selection is completed by identifying multiple focal areas in a given region, typically urban in nature, which sharply contrasts with the study’s independent variables (e.g., high versus low category groups). These contrasting areas are used as either independent groups containing multiple collection sites or a single collection site where sampling then occurs using the same collection methods at each location so that the results can be appropriately compared between sites. This traditional study design has been used to successfully identify effects of land cover type [28], socioeconomic conditions [29,30], degree of urbanization [31], seasonal change [32], precipitation [33], and interspecific competition [34] on *Aedes* population sizes.

While these studies have inarguably provided useful conclusions, there are limitations in their applicability to regional vector control programs. Namely, the sites sampled in these types of studies are often chosen to maximize differences in particular conditions rather than assessing across the range of conditions between extremes. This approach facilitates the interpreting of results, but even if there are intermediate groups or sites chosen, this technique presents an overly simplistic view of the heterogeneity of urban landscapes. For example, urban regions are not collections of high, medium, and low category neighborhoods, but are instead continuous and complex gradients of environmental, social, economic, and physical characteristics [35,36,37]. Thus, drawing conclusions from sites that divide these gradients into neatly differential groups make it difficult to recommend vector control strategies for areas that do not fit the characteristics of any one group. This limitation is also present in the other approaches that have been applied to vector surveillance including: (i) creation of a grid and surveying properties at each intersect [38,39]; (ii) using randomly selected grid cells [40]; (iii) sampling randomly at selected points within a spectral range [41]; (iv) using intervals along roads [42]; and (v) using prior knowledge of presumed vector hotspots to inform sample site selection [43]. Therefore, the development of alternative sampling schemes that consider the heterogeneous nature of urban landscapes will provide a pragmatic tool that improves the efficacy of surveillance.

The goal of this study was to create and evaluate a novel sampling design for *Aedes* mosquitoes which accounts for the complexity of their urban habitats, namely the socioeconomic and environmental variation present in a large urban region. Understanding *Aedes* ecology within the context of social determinants of health will aid in the identification of risk pools within a diverse urban population, allowing vector control programs to maximize their effectiveness by targeting regions or neighborhoods where vector abundance and virus transmission risk is predicted to be highest. Thus, the specific objective of our study is to design a site selection scheme, which objectively and quantifiably targets the widest and most accurate range of socioeconomic and environmental conditions along a continuous covariate gradient. Additionally, we have maximized the distance between each site allowing for the widest geographic coverage of observation and maximizing resource utilization. We present this design as an alternative surveillance method to existing strategies through description of a case study for *Ae. albopictus* surveillance in Mecklenburg County, North Carolina.

## 2. Methods

### 2.1. Study Site

**Figure 1:**
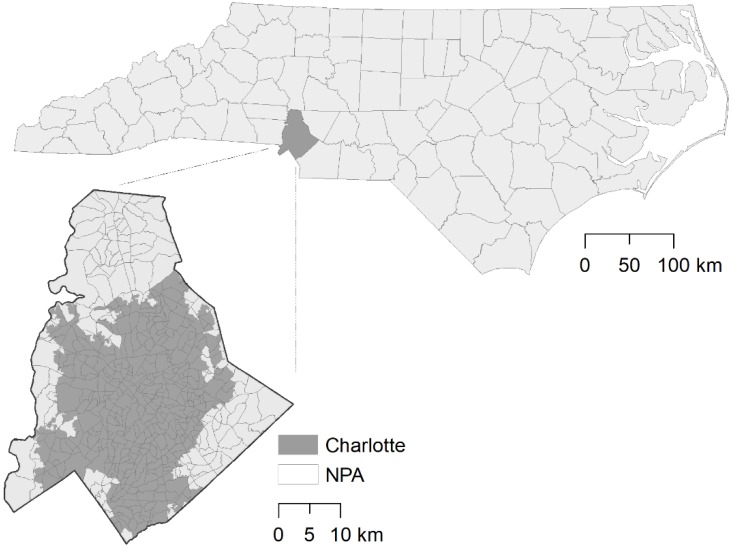
Location of Mecklenburg County and Charlotte city limits in North Carolina (NPA = Neighborhood Planning Area).

Charlotte, North Carolina, located in Mecklenburg County (Figure 1), sits in a humid subtropical climate zone. It totals an average of 1100 mL of precipitation annually, fairly evenly distributed across all seasons, though slightly higher in the summer. Temperatures also peak in the summer months between 25 and 30 degrees Celsius [44]. The temperature and precipitation conditions from July to August are highly conducive to mosquito population growth [45]. Charlotte is one of the fastest growing cities in the United States, with a growth rate of 59.6% over the previous decade [46] and the sixth highest increase in population of any city in the country between 2000 and 2012, and the second highest increase between 2010 and 2013 [47]. Socioeconomically, Charlotte has one of the highest rates of poverty and has the single lowest rate of upward mobility of any city in the U.S., with only 4.4% of children raised in a low income bracket likely to transition to a higher income bracket in their lifetime [48]. A high growth rate combined with the lowest rate of upward mobility indicates that economic disparity in Charlotte is high and likely to continue to increase. As evidence, the Gini Index (0.479) indicates that Charlotte has the tenth highest level of income inequality of any city in U.S. and that the percentage of residents in the 95th percentile and 20th percentile of income are growing at the ninth highest rate in the country, while middle income earners are less common in the metropolitan area [49]. Furthermore, as a result of segregation Charlotte has a unique “crescent and wedge” growth pattern, with high earners tending to occupy a single hyper-concentrated “wedge” of the city while lower income residents are distributed throughout the remaining “crescent”. This has led to considerable differences in the infrastructure and zoning of neighborhoods depending on the income of their residents, which in turn has led to considerable variation in the composition and configuration of the landscape present in those neighborhoods [50]. How strong these differences are and their impact on urban mosquito ecology is still unknown. Thus, while neighborhood-level health outcomes and their relationships to resident socioeconomic attributes have been well-documented [51], it is unclear to what degree local ecological factors contribute to social determinants of health.

General and broad scale mosquito surveillance efforts have occurred in Charlotte throughout the previous three decades. The most recent surveys in the summer of 2017 found *Ae. albopictus* larvae in 25 neighborhoods spread across Mecklenburg County. *Aedes aegypti* has not been seen in the county since the mid-1980s, having since been completely displaced by the invasive and superior competitor *Ae. albopictus*. There have been no assessments aimed at comparing neighborhood socioeconomic characteristics with local mosquito populations in the region.

### 2.2. Trap Location Selection

We identified 90 sample sites, a number recommended by an a priori G*Power analysis [52], using a two-step optimization procedure similar to the ones typically used in soil sampling designs [53]. Specifically, the main objective was to maximize the spatial spreading of mosquito sample sites across the study region, while the secondary objective was to sample at locations that would reflect a large range of socioeconomic conditions. The unit for both phases was the neighborhood planning area (NPA), a geographical delineation originally based on the 1990 U.S. Census tracts and updated following the 2010 U.S. Census. The NPA was used by the Charlotte-Mecklenburg Planning Commission to more accurately fit the geographical boundaries of the county’s neighborhoods than the 1990 U.S. Census tracts did (*n* = 462, see Figure 1). The data used in the optimization procedure were extracted from the Charlotte Quality of Life Study (CQOLS; [50]), a resource which has been used for over 20 years in Mecklenburg County to illustrate and describe the quality of life in the county at the NPA scale based on 80 variables. The data describing each variable are drawn from a variety of local sources including: state, county, and municipal governments. The data are categorized under the following structures: economic, environmental, education, engagement, health, housing, safety, and transportation. More information on the specific methods of the CQOLS can be found at: https://mcmap.org/qol/.

**Figure 2:**
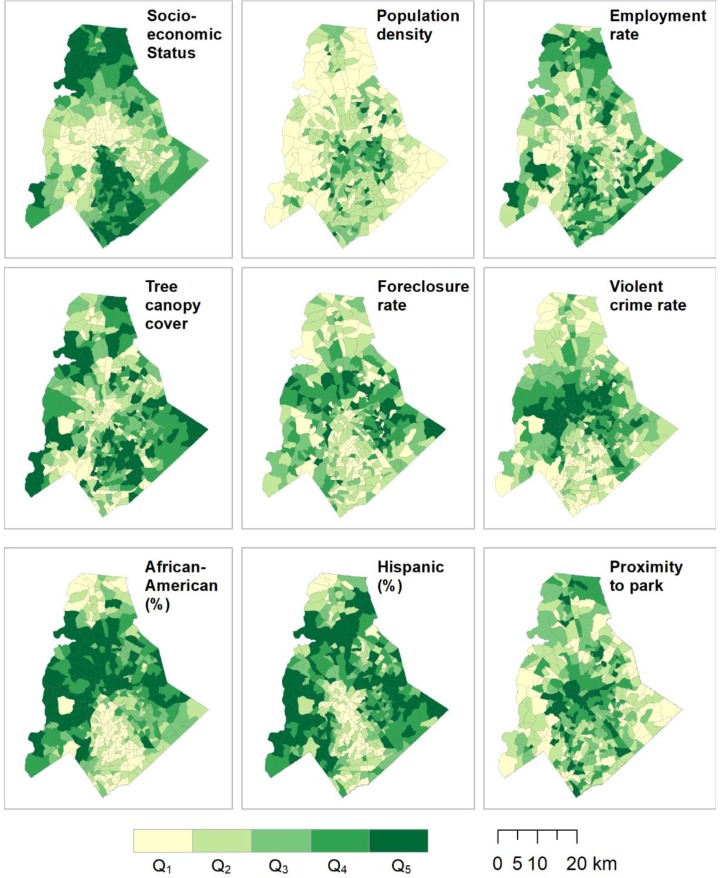
These nine variables, broken into quintiles, were used in the optimization process to identify NPAs suitable for surveying.

We utilized nine variables from the CQOLS, each with a demonstrated or hypothetical relationship with *Aedes* distribution [39,54,55]. These nine variables were selected following a correlation matrix that identified collinearity between one of more of the initially chosen 21 variables. The nine final variables were: socioeconomic status (an index of the normalized weighted average of three common socioeconomic variables—percent with bachelor’s degree, household income, and home sales price); population density; employment rate; total area covered by tree canopy; foreclosure rate; violent crime rate; Hispanic population rate; African-American population rate; and proximity to a park (Figure 2). All variables were standardized from 0 to 1.

### 2.3. Optimization Phase 1: P-dispersion

The first phase of the optimization was aimed at dispersing sampling sites to guarantee wide spatial coverage of study. The sites used for the optimization were the centroids of each NPA. One model that attempts at spreading sites as from one another is the *p*-dispersion model [56], which essentially maximizes the distance that separates any two sites. Four NPAs were excluded from the analysis: two which comprise an airport, and two which did not contain any residential units. The *p*-dispersion model is formulated as follows:

(1) Max D

Subject to:

(2) $$D+\left(M-d_{ij}\right)X_{i}+\left(M-d_{ij}\right)X_{j}\leq 2M-d_{ij}\;\forall i,j>i$$

(3) $$\sum_{i}X_{i}=p$$

(4) $$X_{i}\in \left\{0,1\right\}\;\forall i\in I,j\in J$$

With $X_{i}$ a decision variable equal to 1 when we selected a sample site, located in NPA *i*, and 0 otherwise. The term *d_ij_* is the distance that separates two sample site *i* and *j* and can be calculated prior to optimization. In our model, Equation (1) maximizes the distance *D* between the closest pair of NPAs *i* and *j*. Constraint (2) tracks the distance between NPA centroids, when both are selected (If either NPA *i* or *j* has not been selected, then *D* is forced to be less than or equal to *d_ij_* + *M*, where *M* is a very large number). Constraint (3) stipulates that the total number of sample sites must be equal to *p* (here, *p* = 30). Constraints (4) are binary integer constraints. The advantage of this model is that inter-site distances are tracked.

### 2.4. Optimization Phase 2: Maximal Coverage Approach

**Figure 3:**
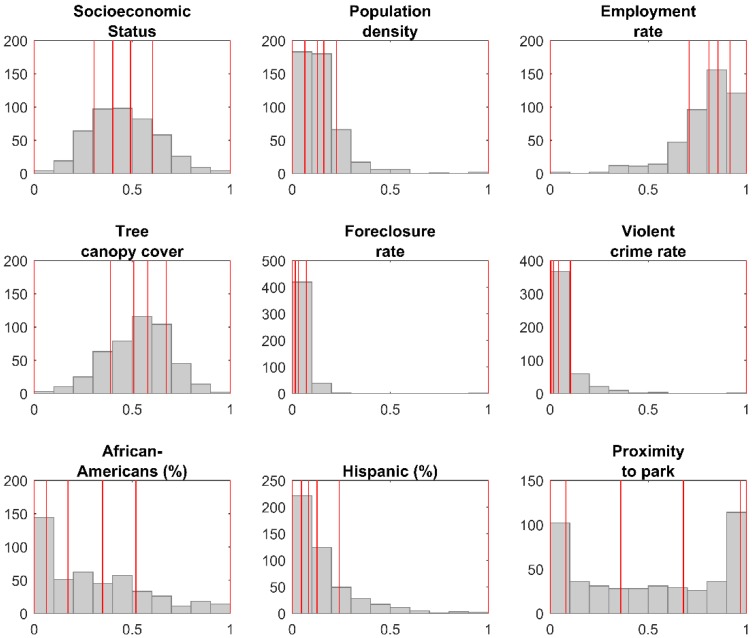
Histogram for each of nine variables used in the optimization process, using all NPAs. The red lines indicate the limits of each quintile.

**Figure 4:**
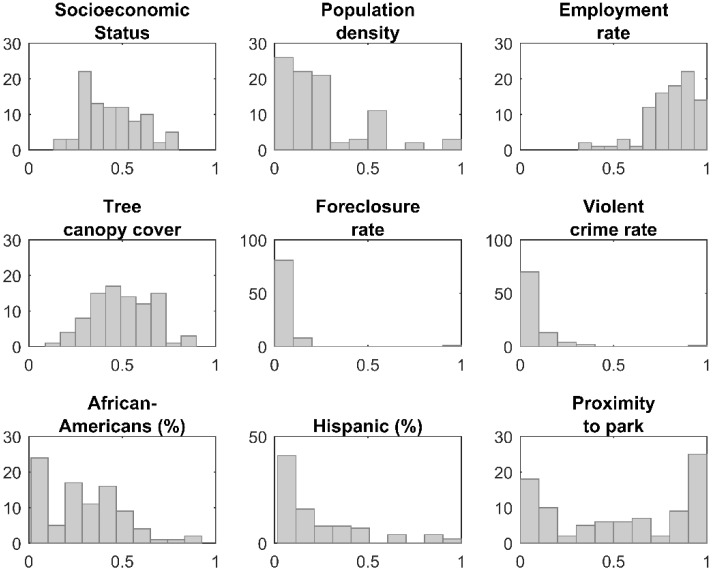
Histogram for each of nine variables using the 90 sites selected in the optimization process (x-axis: standardized variable value; y-axis: count).

For this phase, we added 60 sample sites to the existing set, following a maximal coverage approach [57]. This second phase of the optimization was designed to locate samples in highly populated neighborhoods, and across the widest range of neighborhood input variables. This was done to maximize the potential host population in the study region, because the species of interest in the research, *A. albopictus*, is a peri-domestic urbanized organism that thrives in areas of high human habitation [27,58]. In addition to selecting NPAs with the highest populations, the model is constrained so that the selected NPAs are representative of the data distribution of nine input variables from the CQOLS. For each of the nine aforementioned variables, we identified the 20th, 40th, 60th, 80th, and 100th percentiles (Figure 3). We then constrained the model so that in the final set of 90 neighborhoods (60 + 30), there would be at least 5 NPAs from the 0 to 20th percentile range of each variable, four neighborhoods from the 20th to 40th percentile range for each variable, and so on (Figure 2, Figure 3 and Figure 4; Table 1). Table 2 indicates the results of a Kolmogorov-Smirnov test comparing the distribution of the nine variables with exhaustive and optimized sample, respectively. The coverage approach model is formulated as follows:

(5) $$Max\;\sum_{j\in J}h_{j}Y_{j}$$

Subject to:

(6) $$\sum_{i\in N_{j}}X_{i}\geq Y_{j}$$

(7) $$\sum_{i}X_{i}=p$$

(8) $$\sum_{i}X_{i}\geq 5\;\forall q\in Q,\;k\in K$$

(9) $$X_{i}\in \left\{0,1\right\},\;\forall i\in I$$

(10) $$Y_{j}\in \left\{0,1\right\},\;\forall j\in J$$

**Figure 5:**
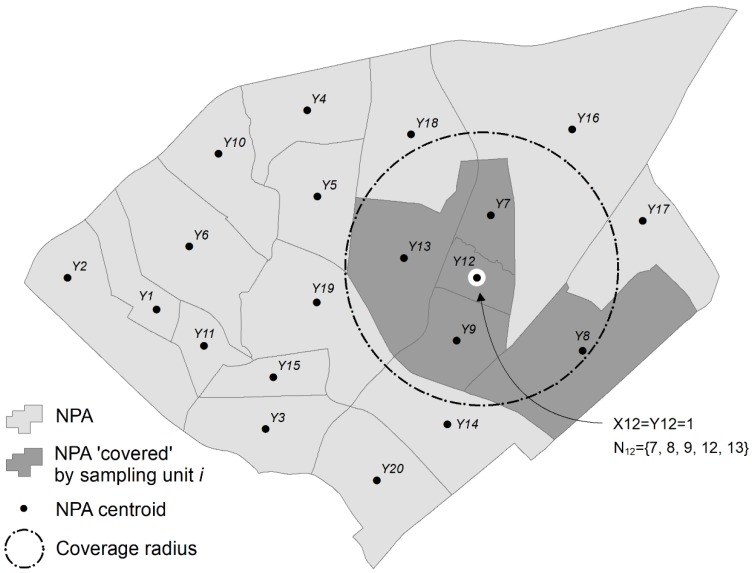
Illustration of Phase 2 mechanism in the optimization procedure. If a sampling unit at *i* = 12 is selected, it will “cover” neighborhoods *j* = 7, 8, 9, 12 and 13.

Whereas $Y_{j}$ is a decision variables equal to 1 when a NPA centroid is “covered” by a sampling site *I* (Figure 5). Whether a sampling site is selected is unknown, hence similar to the *p*-dispersion model, $X_{i}$ needs to be determined through the optimization method. The objective of the model, constraint (5), is to maximize the population $h_{j}$ within NPA *j* that is covered (represented) by the sampling location at *i* (we assume that a population is covered if it is within a “service” distance of 1000 meters from the sample site; this parameter can be modified, but reflects the average diameter of NPAs; Figure 5). As such, samples were located in regions with higher population counts.

Constraint (6) stipulates that an NPA j is covered only when there is at least one sample location *i* in the vicinity of *j*. The latter is defined by imposing a radius around sampling unit *i* and computing the set of NPAs $N_{j}$ potentially covered by sampling unit *I* (Figure 5). Constraint (7) restricts the number of sample sites to be equal to *p* (*p* = 60 in this case). Constraint (8) stipulates that a minimum number of sample locations (here 5) should be selected for each quintile Q (|Q| = 5) of each of the standardized variables K with |K| = 9 (a higher number of samples per quintile made the problem infeasible). Finally, constraints (9) and 10 are standard integrality constraints; sampling sites are located or not in (9) and NPAs are covered or not in (10). Both models were solved using the optimal solver CPLEX [59].

**Figure 6:**
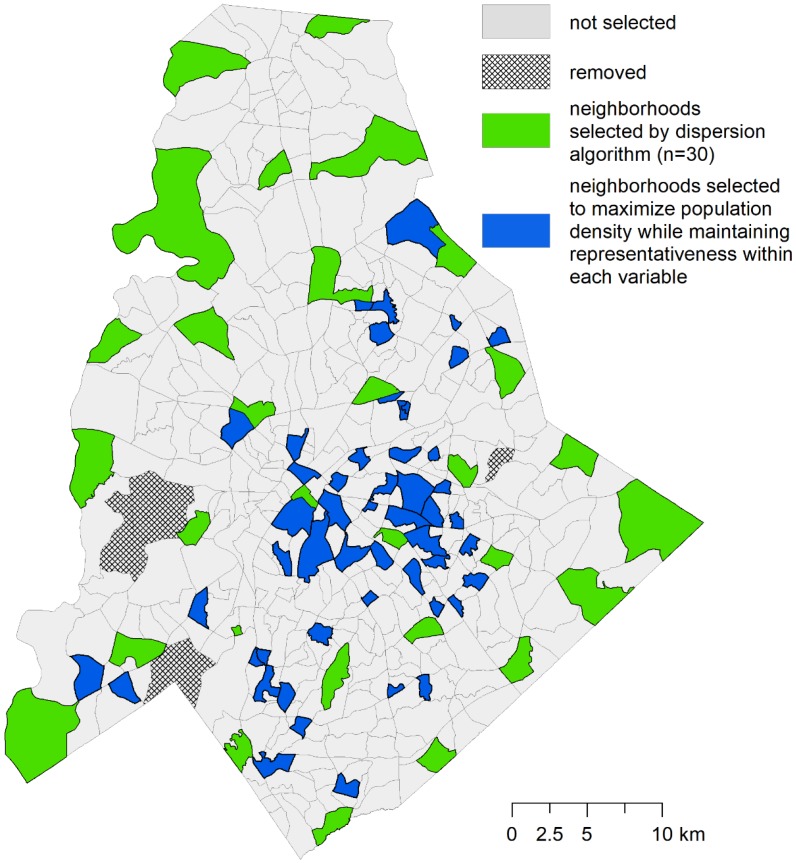
Location of selected NPAs.

Following the selection of 90 samples sites (Figure 6; *n* = 30 from the *p*-dispersion phase and *n* = 60 from the coverage approach phase), we attempted to establish a trap site as close to the NPA’s centroid as possible. If the centroid was located on private property, we asked the property landowner for permission, and if denied, we continued asking the owners of adjacent properties until we were granted access. Once placed, traps were given a unique ID code and georeferenced using a handheld GPS unit. Traps were also marked with a notice dissuading tampering.

### 2.5. Entomological Surveys

Gravid *Aedes* Traps (GAT) were placed at each site selected using the optimization algorithm outlined above. Traps were located in shaded conditions protected from precipitation and direct sunlight. The GAT traps the gravid female as she arrives to lay her eggs in the water-filled container located below a mesh lining [13]. This is counter to host-seeking traps, such as the BG Sentinel [60,61,62], that attract females by mimicking host-characteristics rather than oviposition sites. Host-seeking traps are highly effective yet provide a less direct link to virus transmission potential than gravid traps, which only trap females that have already had a bloodmeal. Additionally, at less than $20 USD per unit, GATs are cost-effective and allow for wider distribution without sacrificing efficacy, compared to host-seeking traps which can cost hundreds of dollars. Additionally, GATs have outperformed other gravid ovitraps, including sticky traps [17,63]. We infused the traps with hay and replaced the infusion at the four and eight week mark across the 12-weeks sample period. We emptied the traps’ contents and identified them [64] in the lab on a weekly basis from 26 May 2017 to 21 August 2017. Highly degraded or rubbed samples were verified to species using PCR techniques conducted at the Walter Reed Biosystematic Unit. This time frame represents the summer months in Charlotte and high season for mosquito activity, where the average daytime high temperature is approximately 29 degrees and the average precipitation is around 90 mm per month [65]. Data was recorded as the number of *Ae. albopictus* females in each trap for each respective week.

### 2.6. Data Analysis

We used a generalized linear model (GLM) with a Poisson distribution and log link, treating weeks as a random effect in a mixed model (GLM; [66]) as well as cross-validation to validate the relationship between the abundance of gravid *Ae. albopictus* and the socioeconomic attributes of the NPA. GLMs have been used numerous times in vector epidemiological research and represent a robust method of variable association for presumed linear relationships [38,67,68,69,70]. We also generated land cover variables based on the heterogeneity of the landscape around each trap site to include in the model as predictors. Specifically, we used land cover maps and the software FRAGSTATS [71] to determine the Shannon land cover diversity and percent covered by each land cover type (tree canopy, grass/shrub, building, road/railroad, other paved surface, and water) within a 30 m radius of each trap site, chosen based on previous research into the average radius of urban *Aedes* hotspots [22,72,73]. Because these values were computed after the optimization process and required us to know the exact location of each trap, we ran an initial GLM model only containing the nine variables used for the second-phase of the optimization process and followed that with a model which included the land cover variables as well, comparing them using Akaike Information Criterion (AIC) values and residuals.

Thus, independent variables included in the second model were the variables at the NPA scale included in the optimization, plus the Shannon land cover diversity and percentage of each land cover type at the 30 m radius scale, while the dependent variable for both models was represented by the abundance of gravid *Ae. albopictus* caught each week at each trap. We ran and validated the model using the Crossfold module in Stata [74], where *k*-fold cross-validation is performed to determine a model’s ability to fit out-of-sample data. This involves splitting the data randomly into *k* partitions (five being the default), then for each partition fitting the specified model using the other *k*-1 groups. The resulting parameters are used to predict the dependent variable in the unused group. Finally, the module reports the root mean squared error (RMSE) for each attempt, with the specified model being validated when the RMSE variation across attempts is minimal.

## 3. Results

**Figure 7:**
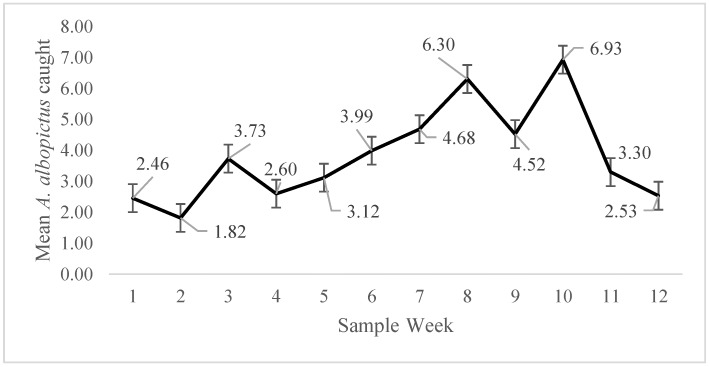
Average number of gravid *Ae. albopictus* caught each week from 26 May 2017 to 21 August 2017.

Following 12 weeks of sampling that took place in each selected NPA, a total of 3,645 gravid female *Ae. albopictus* were collected throughout the length of the study period (Figure 7), with 72% of the traps across all locations and weeks being positive for *Ae. albopictus*. The *Ae. albopictus* represented 86% of the total mosquitoes collected, with the remainder divided between *Ae. trisariatus* (*n* = 203), *Ae. vexans* (*n* = 41), *Ae. japonicus* (*n* = 39), *Culex restuans* (*n* = 41), and *Cx. pipiens* (*n* = 16). These are all known to be common species in the Southeastern U.S. The complete absence of *Ae. aegypti* is not surprising given the recent invasion of *Ae. albopictus* [34]. This result validates Mecklenburg County government surveillance efforts from previous years, as *Ae. aegypti* has not been found locally since the 1980s. There was also a seasonal component, with the number of samples increasing slowly from late May to the height in late July before dropping sharply into August.

**Figure 8:**
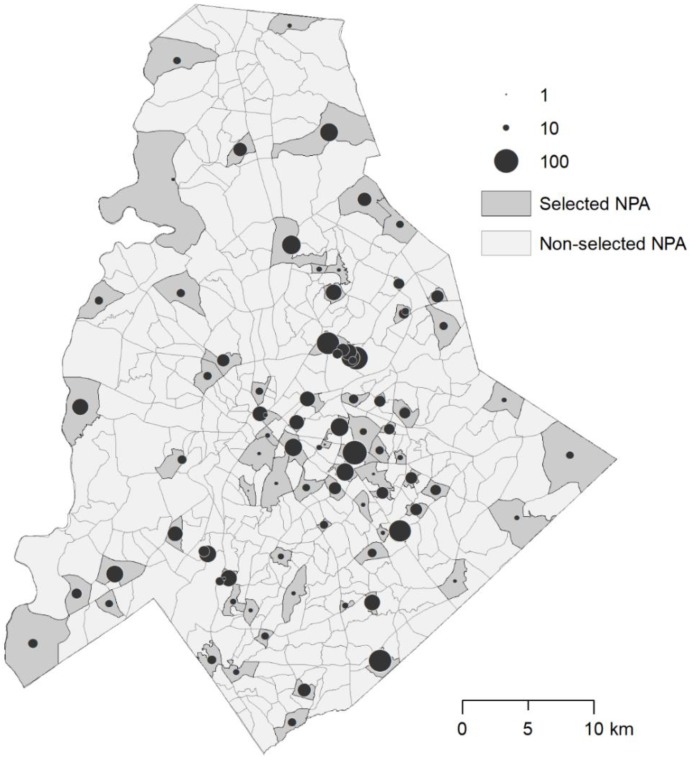
Sum of gravid *A. albopictus* caught over 12 weeks in each selected NPA (small circle: fewer total samples; large circle: more total samples).

The first model (Table 3; AIC = 8.008), containing only the significant variables found among the nine used in optimization, was slightly weaker than the second model (Table 4; AIC = 7.858), which included the land cover variables around each trap, though is less than the generally required value of 2 to signify meaningful difference. This indicates that despite being significant predictors, the land cover variables do not add sufficient additional information the model. The results of the second model illustrate that population, socioeconomic percentile, and percent Hispanic residents have a positive effect while foreclosure rate and violent crime rate have a positive effect on the abundance of gravid *Ae. albopictus* (Table 3). Figure 8 illustrates the sum of all gravid *Ae. albopictus* caught at each site. Finally, the residuals of the models did not exhibit any spatial autocorrelation (Moran’s *I* z-score: 0.002) and the models had consistent RSME and pseudo-R^2^ values across five partitions (Table 5).

## 4. Discussion

Our overall goal was to create and evaluate a novel sampling design for *Aedes* mosquitoes which matched the complexity and heterogeneity of their urban habitats. We were successful in this objective, establishing a list of potential sample sites that accounts for the true distribution of socioeconomic and land cover based values that exist across a major urban region, while also promoting the spatial spreading of selected sites. This study design is beneficial in that it treats the potential predictors as a continuous gradient, which is more accurately represents how they exist in reality, rather than in a collection of groupings. We conceived the design with the intention creating a flexible approach that could be adapted to other urban regions and study systems. With the stipulation being that there is available data on neighborhood-scale attributes, the sample size in both optimization phases can be manipulated based on the desired parameters or size of the region. Additionally, following the optimization procedure, any relevant dependent variable can be measured along the devised sample site gradient.

One of the primary limitations of such a study design is the sacrificing of within-group sample size in exchange for maximizing the number of overall sample groups. With only one trap site per NPA, there is certainly potential for error, though we attempted to offset this by including a minimum of five samples per quintile per input variable. Although it was not possible to set a higher minimum number of samples per quintile due to model infeasibility, increasing the number of explanatory variables in the optimization model (|K|) would force us to select a smaller number of samples per percentile. Overall, this design provided a direct contrast to studies where high numbers of traps are distributed across a small number of sample groups. Both designs have inherent flaws, and while resource limitations prevent studies from placing a trap every 30 m across an entire urban region, surveillance authorities should be cognizant of the costs and benefits to both approaches before designing their own programs.

In our study, we created a pool of study sites that incorporated the widest possible range of neighborhood-scale attributes with potential relationships to *Ae. albopictus* distribution, while concurrently maximizing the distance between sites to increase the spatial coverage of the study. In doing so, we were able to create predictions of gravid *Ae. albopictus* abundance across the distribution of values in the input variables. The results indicate that various metrics associated with socioeconomic status are related to the abundance of gravid *Ae. albopictus*. However, our validation metrics indicate that overall, the model is not highly predictive. This is not unexpected, as mosquito ecology is highly complex, with environmental components such as vegetation, microclimate, and breeding habitat availability likely playing greater roles in the distribution of individuals than neighborhood scale metrics. Still, while other studies have found that *Ae. albopictus* infestation is related to urban decay, resident income, and local resident knowledge [54,75,76,77], this is the first time that the additional socioeconomic metrics of foreclosure rate, violent crime rate, and an index incorporating education, home sales price, and household income have been connected to gravid vector abundance. However, it is unclear how exactly some of these variables are linked to mosquito ecology. While we did not find correlations between independent variables, there may be certain ecological features important to *Ae. albopictus* that link these socioeconomic variables to each other. For example, future studies may investigate relationships between violent crime rate and local abundance of abandoned or dilapidated structures, which may subsequently harbor mosquito breeding habitat. This can aid public health risk assessments by isolating relationships between socioeconomic variables that may directly influence disease risk in a community. Since *Ae. albopictus* breeds in water-filled man-made containers, these socioeconomic variables would seemingly have some positive relationship on breeding habitat availability. This was found in Bakersfield, California [78], where housing delinquency following economic downturn led to neglected swimming pools and other unmanaged water-collecting containers. Similarly, the accumulation of waste in India [79] and Texas [80] as well as the presence of dilapidated urban structures in low-income city blocks in Washington D.C. [81] have been found to be associated with increased vector breeding habitat. However, the relationship is not consistent. For example, in Baltimore in 2014, Becker et al. found that mosquito production was higher in high SES neighborhoods than low SES neighborhoods, hypothesized to be related to containers being regularly yet artificially supplied with water, while unmanaged rain-filled containers in low SES neighborhoods dry up too quickly to be utilized for breeding. In general, while vector ecology has been well-studied, we suggest that future studies focus more diligently on conditions in the host community that may support ecological underpinnings. This not only includes examinations of socioeconomic drivers of vector abundance, but can also involve studies of resident knowledge of risk or variations in vector control practices.

Overall, these studies hold important implications for public health, as exposure to vector-borne diseases can be viewed under the lens of health disparities. Communities with lower relative educational attainment [82], poor economic stability [83], and lower property values [84] are known to be at a disproportionate risk of a myriad of health concerns. Our findings indicate that higher exposure to vector-borne diseases is an additional risk factor that can further erode the health of these already disadvantaged community members. Indeed, relationships between socioeconomic or demographic predictors and mosquito-borne disease risk have been established for West Nile virus [85,86,87], St. Louis encephalitis [88], and dengue virus [89,90].

As previous studies did not utilize GATs, our results provide indication that while gravid *Ae. albopictus* abundance is higher in neighborhoods of lowered socioeconomic status and high landscape diversity, human biting rate may also be higher in such neighborhoods. Our traps solely captured females who have consumed a bloodmeal, and while *Ae. albopictus* host preference is more diverse than *Ae. aegypti* [91], studies in urban areas indicate that humans still comprise 80–100% of *Ae. albopictus* host targets [7,9]. Further studies specifically focused on biting rates and host preferences would be needed to confirm this hypothesis.

Additionally, the determination that gravid *Ae. albopictus* abundance is higher in neighborhoods with high proportions of residents that identify as Hispanic is the first known published relationship between gravid vector abundance and local ethnicity. Additional studies would be needed to determine how variables correlated with particular ethnicities are related to vector ecology. Knowledge, attitude, and practice surveys [92,93,94] in potentially at-risk communities may be a useful method of understanding how cultural practices can lead to increased contact with host-seeking *Ae. albopictus*. Regardless, with higher gravid *Ae. albopictus* abundance than sites with other predominant ethnicities, we suggest that public health authorities take particular care to address mosquito-biting risk in the Hispanic community, including designing specialized bilingual public education or information campaigns as-needed.

The relationship between vector abundance and land cover has been studied in diverse regions including Thailand [95], Hawaii [96], South Dakota [97], Peru [98], and Chicago [99]. Our demonstration that land cover variables have no meaningful effect on a model which already includes socioeconomic variables may indicate the reduced scale of impact of landscape metrics on gravid *Ae. albopictus* abundance compared to other neighborhood characteristics. However, as land use change continues into suburban and rural areas, increased vector suitability and potential for virus transmission may be a consequence of such development [100,101] and municipal vector control efforts should increase proportionally.

It is important to note that while we have indeed illustrated key relationships between socioeconomic variables and gravid *Ae. albopictus* abundance, there are numerous other variables we did not examine with hypothesized or proven links to *Ae. albopictus* ecology. In future surveys, combining socioeconomic attributes with other meaningful independent variables such as climate, interspecific-competition, predation, and vector control would reduce missing variable bias and aid in building a more comprehensive picture of vector distribution and potential virus transmission risk.

## 5. Conclusions

Our study provides a novel vector surveillance approach which accounts for the true distribution of socioeconomic attribute values in an urban region. We intend for this design to be compared and contrasted with existing surveillance approaches so that authorities can maximize the validity of their results. In our study, we used socioeconomic variation across the urban region as the central focus of the optimization, though there is potential to use any other data that may be available at the neighborhood scale to identify ideal sample sites, including environmental, physical, social, or cultural neighborhood attributes. We found that gravid *Ae. albopictus* abundance is negatively related to several neighborhood socioeconomic characteristics and demonstrate the importance of understanding vector-borne disease risk within the context of social determinants of health. In general, the body of literature examining *Aedes* ecology and urban socioeconomic variation is small yet growing, and further investigation will be required to better understand the nature and implications of this important relationship.

## Figures and Tables

**Table 1:** Quintile ranges for each of the nine variables used in the optimization process and number of sample points within each range.

Variable	Q1	Q2	Q3	Q4	Q5
Socioeconomic Status	[0–0.305)	[0.305–0.401)	[0.401–0.491)	[0.491–0.603)	[0.603–1)
	19	22	16	16	17
Population density	[0–0.0645)	[0.0645–0.129)	[0.129–0.161)	[0.161–0.225)	[0.225–1)
	18	18	5	15	34
Employment rate	[0–0.708)	[0.708–0.810)	[0.810–0.856)	[0.856–0.916)	[0.916–1)
	20	16	18	22	14
Tree canopy cover	[0–0.388)	[0.388–0.508)	[0.508–0.579)	[0.579–0.674)	[0.674–1)
	23	22	16	14	15
Foreclosure rate	[0–0.016)		[0.016–0.032)	[0.032–0.072)	[0.072–1)
	43		15	18	14
Violent crime rate	[0–0.005)	[0.005–0.017)	[0.017–0.043)	[0.043–0.103)	[0.103–1)
	15	15	19	24	17
African Americans (%)	[0–0.062)	[0.062–0.172)	[0.172–0.349)	[0.349–0.519)	[0.519–1)
	11	18	28	20	13
Hispanic (%)	[0–0.047)	[0.047–0.083)	[0.083–0.127)	[0.127–0.239)	[0.239–1)
	18	16	13	12	31
Proximity to park	[0–0.08)	[0.08–0.36)	[0.36–0.68)	[0.68–0.97)	[0.97–1)
	17	16	20	17	20

**Table 2:** Results from the Kolmogorov-Smirnov test comparing the distribution of the nine variables with exhaustive and optimized sample, respectively. The *p*-value indicates the significance level.

Variable	(at 1%)	
	*t*	*p*
Socioeconomic Status	0	0.4493
Population Density	1	0.0001
Employment Rate	0	0.9074
Tree Canopy	0	0.2081
Foreclosure Rate	0	0.9950
Violent Crime Rate	0	0.4321
Percent African American	0	0.4374
Percent Latino	0	0.0488
Proximity to Park	0	0.9652

**Table 3:** Results of the first GLM predicting the abundance of gravid *Ae. Albopictus*.

Independent Variable	Source	Coefficient	*p*
Housing Density	CQOLS	−0.021	<0.01
Socioeconomic percentile	CQOLS	−0.731	<0.01
Foreclosure rate	CQOLS	0.050	<0.01
Violent crime rate	CQOLS	0.005	<0.01
Percent of residents Hispanic	CQOLS	0.007	<0.01

**Table 4:** Results of the second GLM predicting the abundance of gravid *Ae. Albopictus*.

Independent Variable	Source	Coefficient	*p*
Housing Density	CQOLS	−0.107	<0.01
Socioeconomic percentile	CQOLS	−2.274	<0.01
Foreclosure rate	CQOLS	0.241	<0.01
Violent crime rate	CQOLS	0.039	<0.01
Percent of residents Hispanic	CQOLS	0.033	<0.01
Percent of land covered by buildings	Mecklenburg County GIS	−4.938	<0.01
Percent of land covered by roads/railroads	Mecklenburg County GIS	−11.98	<0.01
Percent of land covered by grass/shrubs	Mecklenburg County GIS	−5.681	<0.01
Percent of land covered by tree canopy	Mecklenburg County GIS	−2.698	<0.01
Shannon diversity index: land cover types	Mecklenburg County GIS	1.718	<0.01

**Table 5:** RMSE as calculated by Crossfold model validation.

Model Run	RMSE	Psuedo-R^2^
1	5.354	0.075
2	4.744	0.048
3	4.142	0.053
4	5.112	0.062
5	4.891	0.044

## References

[1] Dash A.P., Bhatia R., Sunyoto T., Mourya D.T. (2013). Emerging and re-emerging arboviral diseases in Southeast Asia. J. Vector Borne Dis..

[2] Gubler D.J. (2002). The global emergence/resurgence of arboviral diseases as public health problems. Arch. Med. Res..

[3] Vasconcelos P.F.C., Calisher C.H. (2016). Emergence of Human Arboviral Diseases in the Americas, 2000–2016. Vector-Borne Zoonotic Dis..

[4] Romanović M., Zorić I. (2006). Breeding habitat diversity of medically important mosquitoes and the risk of spreading exotic species in the coastal area of Croatia. Period. Biol..

[5] Seng C.M., Jute N. (1994). Breeding of *Aedes aegypti* (L.) and *Aedes albopictus* (Skuse) in urban housing of Sibu town, Sarawak. Southeast Asian J. Trop. Med. Public Health.

[6] Farjana T., Tuno N. (2013). Multiple blood feeding and host-seeking behavior in *Aedes aegypti* and *Aedes albopictus* (Diptera: Culicidae). J. Med. Entomol..

[7] Ponlawat A., Harrington L.C. (2005). Blood Feeding Patterns of *Aedes aegypti* and *Aedes albopictus* in Thailand. J. Med. Entomol..

[8] Buckner E.A., Blackmore M.S., Golladay S.W., Covich A.P. (2011). Weather and landscape factors associated with adult mosquito abundance in southwestern Georgia, USA. J. Vector Ecol..

[9] Richards S.L., Ponnusamy L., Unnasch T.R., Hassan H.K., Apperson C.S. (2006). Host-feeding patterns of *Aedes albopictus* (Diptera: Culicidae) in relation to availability of human ad domestic animals in suburban landscapes of Central North Carolina. J. Med. Entomol..

[10] Morin C.W., Comrie A.C., Ernst K. (2013). Climate and dengue transmission: Evidence and implications. Environ. Health Perspect..

[11] Charrel R.N., de Lamballerie X., Raoult D. (2007). Chikungunya Outbreaks—The Globalization of Vectorborne Diseases. N. Engl. J. Med..

[12] Tauil P.L. (2001). Urbanization and dengue ecology. Cadernos de Saúde Pública.

[13] Eiras A.E., Buhagiar T.S., Ritchie S.A. (2014). Development of the Gravid *Aedes* Trap for the Capture of Adult Female Container-Exploiting Mosquitoes (Diptera: Culicidae). J. Med. Entomol..

[14] Meeraus W.H., Armistead J.S., Arias J.R. (2008). Field Comparison of Novel and Gold Standard Traps for Collecting Aedes albopictus in Northern Virginia. J. Am. Mosq. Cont. Control Assoc..

[15] Norzahira R., Hidayatulfathi O., Wong H.M., Cheryl A., Firdaus R., Chew H.S., Lim K.W., Sing K.W., Mahathavan M., Nazni W.A. (2011). Ovitrap surveillance of the dengue vectors, *Aedes* (Stegomyia) *aegypti* (L.) and *Aedes* (Stegomyia) *albopictus* skuse in selected areas in Bentong, Pahang, Malaysia. Trop. Biomed..

[16] Wong J., Bayoh N., Olang G., Killeen G.F., Hamel M.J., Vulule J.M., Gimnig J.E. (2013). Standardizing operational vector sampling techniques for measuring malaria transmission intensity: evaluation of six mosquito collection methods in western Kenya. Malar. J..

[17] Ritchie S.A., Buhagiar T.S., Townsend M., Hoffmann A., Van Den Hurk A.F., McMahon J.L., Eiras A.E. (2014). Field validation of the gravid *Aedes* trap (GAT) for collection of *Aedes aegypti* (Diptera: Culicidae). J. Med. Entomol..

[18] Facchinelli L., Valerio L., Pombi M., Reiter P., Costantini C., Della Torre A. (2007). Development of a novel sticky trap for container-breeding mosquitoes and evaluation of its sampling properties to monitor urban populations of Aedes albopictus. Med. Vet. Entomol..

[19] Dia I., Diallo D., Duchemin J.-B., Ba Y., Konate L., Costantini C., Diallo M. (2005). Comparisons of human-landing catches and odor-baited entry traps for sampling malaria vectors in Senegal. J. Med. Entomol..

[20] Hii J.L.K., Smith T., Mai A., Ibam E., Alpers M.P. (2000). Comparison between anopheline mosquitoes (Diptera: Culicidae) caught using different methods in a malaria endemic area of Papua New Guinea. Bull. Entomol. Res..

[21] Degener C.M., Azara T., Roque R.A., Codeco C.T., Nobre A.A., Ohly J.J., Geier M., Eiras A.E. (2014). Temporal abundance of *Aedes aegypti* in Manaus, Brazil, measured by two trap types for adult mosquitoes. Mem. Inst. Oswaldo Cruz.

[22] Chansang C., Kittayapong P. (2007). Application of mosquito sampling count and geospatial methods to improve dengue vector surveillance. Am. J. Trop. Med. Hyg..

[23] Silver J.B. Mosquito Ecology—Field Sampling Methods, Third Edition. http://www.springer.com/gp/book/9781402066658.

[24] Morrison A.C., Astete H., Chapilliquen F., Ramirez-Prada C., Diaz G., Getis A., Gray K., Scott T.W. (2004). Evaluation of a sampling methodology for rapid assessment of *Aedes aegypti* infestation levels in Iquitos, Peru. J. Med. Entomol..

[25] Sanchez L., Cortinas J., Pelaez O., Gutierrez H., Concepción D., Van Der Stuyft P. (2010). Breteau Index threshold levels indicating risk for dengue transmission in areas with low *Aedes* infestation. Trop. Med. Int. Heal..

[26] Vezzani D., Albicócco A.P. (2009). The effect of shade on the container index and pupal productivity of the mosquitoes *Aedes aegypti* and *Culex pipiens* breeding in artificial containers. Med. Vet. Entomol..

[27] Saleeza S.N.R., Norma-Rashid Y., Sofian-Azirun M. (2011). Mosquitoes Larval Breeding Habitat in Urban and Suburban Areas, Peninsular Malaysia. Int. J. Biol. Vet. Agric. Food Eng..

[28] Bartlett-Healy K., Unlu I., Obenauer P.J., Hughes T.H., Healy S.P., Crepeau T.N., Farajollahi A., Kesavaraju B., Fonseca D.M., Schoeler G. (2012). Larval mosquito habitat utilization and community dynamics of *Aedes albopictus* and *Aedes japonicus* (Diptera: Culicidae). J. Med. Entomol..

[29] Becker B., Leisnham P.T., LaDeau S.L. (2014). A tale of two city blocks: Differences in immature and adult mosquito abundances between socioeconomically different urban blocks in Baltimore (Maryland, USA). Int. J. Environ. Res. Public Health.

[30] LaDeau S.L., Leisnham P.T., Biehler D., Bodner D. (2013). Higher mosquito production in low-income neighborhoods of Baltimore and Washington, DC: Understanding ecological drivers and mosquito-borne disease risk in temperate cities. Int. J. Environ. Res. Public Health.

[31] Tsuda Y., Suwonkerd W., Chawprom S., Prajakwong S., Takagi M. (2006). Different spatial distribution of *Aedes aegypti* and *Aedes albopictus* along an urban-rural gradient and the relating environmental factors examined in three villages in northern Thailand. J. Am. Mosq. Control Assoc..

[32] Rozilawati H., Zairi J., Adanan C.R. (2007). Seasonal abundance of *Aedes albopictus* in selected urban and suburban areas in Penang, Malaysia. Trop. Biomed..

[33] Moore C.G., Cline B.L., Ruiz-Tiben E., Lee D., Romney-Joseph H., Rivera-Correa E. (1978). *Aedes aegypti* in Puerto Rico: Environmental determinants of larval abundance and relation to dengue virus transmission. Am. J. Trop. Med. Hyg..

[34] Bagny Beilhe L., Arnoux S., Delatte H., Lajoie G., Fontenille D. (2012). Spread of invasive *Aedes albopictus* and decline of resident *Aedes aegypti* in urban areas of Mayotte 2007-2010. Biol. Invasions.

[35] Pickett S.T.A., Cadenasso M.L., Grove J.M., Boone C.G., Groffman P.M., Irwin E., Kaushal S.S., Marshall V., McGrath B.P., Nilon C.H. (2011). Urban ecological systems: Scientific foundations and a decade of progress. J. Environ. Manag..

[36] Salat S., Bourdic L., Nowacki C. (2010). Assessing Urban Complexity. Int. J. Sustain. Build. Technol. Urban Dev..

[37] Rydin Y., Bleahu A., Davies M., Dávila J.D., Friel S., De Grandis G., Groce N., Hallal P.C., Hamilton I., Howden-Chapman P. (2012). Shaping cities for health: Complexity and the planning of urban environments in the 21st century. Lancet.

[38] Carbajo A.E., Curto S.I., Schweigmann N.J. (2006). Spatial distribution pattern of oviposition in the mosquito Aedes aegypti in relation to urbanization in Buenos Aires: southern fringe bionomics of an introduced vector. Med. Vet. Entomol..

[39] Walker K.R., Joy T.K., Ellers-Kirk C., Ramberg F.B. (2011). Human and Environmental Factors Affecting Aedes aegypti Distribution in an Arid Urban Environment. J. Am. Mosq. Control Assoc..

[40] Keating J., Macintyre K., Mbogo C., Githeko A., Regens J.L., Swalm C., Ndenga B., Steinberg L.J., Kibe L., Githure J.I. (2003). A geographic sampling strategy for studying relationships between human activity and malaria vectors in urban Africa. Am. J. Trop. Med. Hyg..

[41] Ageep T.B., Cox J., Hassan M.M., Knols B.G.J., Benedict M.Q., Malcolm C.A., Babiker A., El Sayed B.B. (2009). Spatial and temporal distribution of the malaria mosquito *Anopheles arabiensis* in northern Sudan: influence of environmental factors and implications for vector control. Malar. J..

[42] Delatte H., Dehecq J.S., Thiria J., Domerg C., Paupy C., Fontenille D. (2008). Geographic distribution and developmental sites of *Aedes albopictus* (Diptera: Culicidae) during a Chikungunya epidemic event. Vector Borne Zoonotic Dis..

[43] Unlu I., Farajollahi A., Healy S.P., Crepeau T., Bartlett-Healy K., Williges E., Strickman D., Clark G.G., Gaugler R., Fonseca D.M. (2011). Area-wide management of *Aedes albopictus*: Choice of study sites based on geospatial characteristics, socioeconomic factors and mosquito populations. Pest Manag. Sci..

[44] Southeast Regional Climate Center. http://www.sercc.com/.

[45] Kraemer M.U.G., Sinka M.E., Duda K.A., Mylne A., Shearer F.M., Barker C.M., Moore C.G., Carvalho R.G., Coelho G.E., Van Bortel W. (2015). The global distribution of the arbovirus vectors *Aedes aegypti* and *Ae. albopictus*. Elife.

[46] United States Census Bureau Population and Housing Unit Estimates 2010. https://www.census.gov/programs-surveys/popest.html.

[47] Cohen D.T., Hatchard G.W., Wilson S.G. Population trends in incorporated places: 2000 to 2013. https://www.census.gov/content/dam/Census/library/publications/2015/demo/p25-1142.pdf.

[48] Chetty R., Hendren N., Kline P., Saez E. (2014). Where is the land of Opportunity? The Geography of Intergenerational Mobility in the United States. Q. J. Econ..

[49] World Bank (2015). The World Bank GINI Index (World Bank Estimate). https://data.worldbank.org/indicator/si.pov.gini.

[50] Charlotte Quality of Life Explorer 2017. https://mcmap.org/qol/.

[51] United States Department of Health and Human Services (2003). Public Health Service Centers for Disease Control and Prevention (CDC) Atlanta, GA 30333. Prevention.

[52] Faul F., ErdFelder E., Lang A.-G., Buchner A. (2009). Statistical power analyses using G*Power 3.1: Tests for correlation and regression analyses. Behav. Res. Methods.

[53] Delmelle E.M., Goovaerts P. (2009). Second-phase sampling designs for non-stationary spatial variables. Geoderma.

[54] Dowling Z., Armbruster P., Ladeau S.L., Decotiis M., Mottley J., Leisnham P.T. (2013). Linking mosquito infestation to resident socioeconomic status, knowledge, and source reduction practices in Suburban Washington, DC. Ecohealth.

[55] Megahed Y., Cabral P., Silva J., Caetano M. (2015). Land Cover Mapping Analysis and Urban Growth Modelling Using Remote Sensing Techniques in Greater Cairo Region—Egypt. ISPRS Int. J. Geo-Inf..

[56] Kuby M.J. (1987). Programming models for facility dispersion: The p-dispersion and maxisum dispersion problems. Geogr. Anal..

[57] Church R., ReVelle C. (1974). The maximal covering location problem. Pap. Region. Sci.

[58] Little E., Biehler D., Leisnham P.T., Jordan R., Wilson S., Ladeau S.L. (2017). Socio-Ecological Mechanisms Supporting High Densities of *Aedes albopictus* (Diptera: Culicidae) in Baltimore, MD. J. Med. Entomol..

[59] CPLEX I.I. (2009). User’s Manual for CPLEX. Man. CPLEX.

[60] Arimoto H., Harwood J.F., Nunn P.J., Richardson A.G., Gordon S., Obenauer P.J. (2015). Comparison of Trapping Performance Between the Original BG-Sentinel® Trap and BG-Sentinel 2® Trap^1^. J. Am. Mosq. Control Assoc..

[61] Maciel-de-Freitas R., Eiras Á.E., Lourenço-de-Oliveira R. (2006). Field evaluation of effectiveness of the BG-Sentinel, a new trap for capturing adult *Aedes aegypti* (Diptera: Culicidae). Mem. Inst. Oswaldo Cruz.

[62] Lourenço-de-Oliveira R., Lima J.B.P., Peres R., da Costa Alves F., Eiras Á.E., Codeço C.T. (2008). Comparison of Different Uses of Adult Traps and Ovitraps for Assessing Dengue Vector Infestation in Endemic Areas. J. Am. Mosq. Control Assoc..

[63] Mackay A., Amador M., Barrera R. (2013). An improved autocidal gavid ovitrap for the control and surveillance of *Aedes aegypti*. Parasit. Vectors.

[64] Potter M. Identification key to the genera of adult mosquitoes for the world 2017. http://www.wrbu.org/mqID/keysMQZoogeo.html.

[65] NOAA National Weather Forcasting Service. https://w2.weather.gov/climate/index.php?wfo=gsp.

[66] Mccullagh P., Nelder J. (1972). Generalized Linear Models.

[67] Honório N.A., Codeço C.T., Alves F.C., Magalhães M.A.F.M., Lourenço-De-Oliveira R. (2009). Temporal distribution of *Aedes aegypti* in different districts of Rio de Janeiro, Brazil, measured by two types of traps. J. Med. Entomol..

[68] Yoo E.-H. (2014). Site-specific prediction of West Nile virus mosquito abundance in Greater Toronto Area using generalized linear mixed models. Int. J. Geogr. Inf. Sci..

[69] Velo E., Kadriaj P., Mersini K., Shukullari A., Manxhari B., Simaku A., Hoxha A., Caputo B., Bolzoni L., Rosà R. (2016). Enhancement of *Aedes albopictus* collections by ovitrap and sticky adult trap. Parasites Vectors.

[70] Wang J., Ogden N.H., Zhu H. (2011). The Impact of Weather Conditions on *Culex pipiens* and *Culex restuans* (Diptera: Culicidae) Abundance: A Case Study in Peel Region. J. Med. Entomol..

[71] McGarigal K., Cushman S.A., Ene E. FRAGSTATS v4: Spatial Pattern Analysis Program for Categorical and Continuous Maps. Computer Software Program Produced by the Authors at the University of Massachusetts, Amherst. http://www.umass.edu/landeco/research/fragstats/fragstats.html.

[72] LaCon G., Morrison A.C., Astete H., Stoddard S.T., Paz-Soldan V.A., Elder J.P., Halsey E.S., Scott T.W., Kitron U., Vazquez-Prokopec G.M. (2014). Shifting Patterns of *Aedes aegypti* Fine Scale Spatial Clustering in Iquitos, Peru. PLoS Negl. Trop. Dis..

[73] Schafrick N.H., Milbrath M.O., Berrocal V.J., Wilson M.L., Eisenberg J.N.S. (2013). Spatial clustering of *Aedes aegypti* related to breeding container characteristics in coastal Ecuador: Implications for dengue control. Am. J. Trop. Med. Hyg..

[74] Daniels B. CROSSFOLD: Stata Module to Perform K-Fold Cross-Validation. https://econpapers.repec.org/software/bocbocode/s457426.htm.

[75] Quintero J., Carrasquilla G., Suárez R., González C., Olano V.A. (2009). An ecosystemic approach to evaluating ecological, socioeconomic and group dynamics affecting the prevalence of Aedes aegypti in two Colombian towns. Cadernos de Saude Publica.

[76] Ruiz M.O., Tedesco C., McTighe T.J., Austin C., Uriel K. (2004). Environmental and social determinants of human risk during a West Nile virus outbreak in the greater Chicago area, 2002. Int. J. Health Geogr..

[77] Kutz F.W., Wade T.G., Pagac B.B. (2003). A geospatial study of the potential of two exotic species of mosquitoes to impact the epidemiology of West Nile virus in Maryland. J. Am. Mosq. Control Assoc..

[78] Reisen W.K., Takahashi R.M., Carroll B.D., Quiring R. (2008). Delinquent mortgages, neglected swimming pools, and West Nile virus, California. Emerg. Infect. Dis..

[79] Banerjee S., Aditya G., Saha G.K. (2013). Household disposables as breeding habitats of dengue vectors: Linking wastes and public health. Waste Manag..

[80] Farajollahi A., Fonseca D., Kramer L., Kilpatric A.M. (2011). ‘Bird Biting’ mosquitoes and human disease: A review of the role of Culex pipiens complex mosquitoes in epipdemiology. Infect. Genet. Evol..

[81] Dowling Z., Ladeau S.L., Armbruster P., Biehler D., Leisnham P.T., Dowling Z., Ladeau S.L., Armbruster P., Biehler D. (2013). Socioeconomic Status Affects Mosquito (Diptera: Culicidae) Larval Habitat Type Availability and Infestation Level. J. Med. Entomol..

[82] Winkleby M.A., Jatulis D.E., Frank E., Fortmann S.P. (1992). Socioeconomic status and health: How education, income, and occupation contribute to risk factors for cardiovascular disease. Am. J. Public Health.

[83] Kjellstrom T., Friel S., Dixon J., Corvalan C., Rehfuess E., Campbell-Lendrum D., Gore F., Bartram J. (2007). Urban environmental health hazards and health equity. J. Urban Health.

[84] Portney P.R. (1981). Housing prices, health effects, and valuing reductions in risk of death. J. Environ. Econ. Manag..

[85] Rochlin I., Turbow D., Gomez F., Ninivaggi D.V., Campbell S.R. (2011). Predictive mapping of human risk for west nile virus (WNV) based on environmental and socioeconomic factors. PLoS ONE.

[86] Lockaby G., Noori N., Morse W., Zipperer W., Kalin L., Governo R., Sawant R., Ricker M. (2016). Climatic, ecological, and socioeconomic factors associated with West Nile virus incidence in Atlanta, Georgia, U.S.A.. J. Vector Ecol..

[87] Liu H., Weng Q., Gaines D. (2011). Geographic incidence of human West Nile virus in northern Virginia, USA, in relation to incidence in birds and variations in urban environment. Sci. Total Environ..

[88] Rios J., Hacker C.S., Hailey C.A., Parsons R.E. (2006). Demographic and spatial analysis of West Nile virus and St. Louis encephalitis in Houston, Texas. J. Am. Mosq. Control Assoc..

[89] Kikuti M., Cunha G.M., Paploski I.A.D., Kasper A.M., Silva M.M.O., Tavares A.S., Cruz J.S., Queiroz T.L., Rodrigues M.S., Santana P.M. (2015). Spatial distribution of dengue in a Brazilian Urban slum setting: Role of socioeconomic gradient in disease risk. PLoS Negl. Trop. Dis..

[90] Delmelle E., Hagenlocher M., Kienberger S., Casas I. (2016). A spatial model of socioeconomic and environmental determinants of dengue fever in Cali, Colombia. Acta Tropica.

[91] Harrington L.C., Edman J.D., Scott T.W. (2001). Why do female *Aedes aegypti* (Diptera: Culicidae) feed preferentially and frequently on human blood?. J. Med. Entomol..

[92] Ayyamani U.D., Ying G.C., San O.G. (1986). A knowledge attitude and practice (KAP) study on dengue/dengue haemorrhagic fever and the *Aedes* mosquitoes. Med. J. Malaysia.

[93] Hairi F., Ong C.H.S., Suhaimi A., Tsung T.W., Bin Anis Ahmad M.A., Sundaraj C., Soe M.M. (2003). A Knowledge, Attitude and Practices (KAP) Study on Dengue among Selected Rural Communities in the Kuala Kangsar District. Asia-Pacific J. Public Heal..

[94] Launiala A. (2009). How much can a KAP survey tell us about people’s knowledge, attitudes and practices? Some observations from medical anthropology research on malaria in pregnancy in Malawi. Anthropol. Matters J..

[95] Vanwambeke S.O., Somboon P., Harbach R.E., Isenstadt M., Lambin E.F., Walton C., Butlin R.K. (2007). Landscape and land cover factors influence the presence of *Aedes* and *Anopheles* larvae. J. Med. Entomol..

[96] Vanwambeke S.O., Bennett S.N., Kapan D.D. (2011). Spatially disaggregated disease transmission risk: Land cover, land use and risk of dengue transmission on the island of Oahu. Trop. Med. Int. Heal..

[97] Chuang T.-W., Hildreth M.B., Vanroekel D.L., Wimberly M.C. (2011). Weather and Land Cover Influences on Mosquito Populations in Sioux Falls, South Dakota. J. Med. Entomol..

[98] Johnson M.F., Gómez A., Pinedo-Vasquez M. (2008). Land Use and Mosquito Diversity in the Peruvian Amazon. J. Med. Entomol..

[99] Chaves L.F., Hamer G.L., Walker E.D., Brown W.M., Ruiz M.O., Kitron U.D. (2011). Climatic variability and landscape heterogeneity impact urban mosquito diversity and vector abundance and infection. Ecosphere.

[100] Norris D.E. (2004). Mosquito-borne Diseases as a Consequence of Land Use Change. Ecohealth.

[101] Vanwambeke S.O., Lambin E.F., Eichhorn M.P., Flasse S.P., Harbach R.E., Oskam L., Somboon P., Van Beers S., Van Benthem B.H.B., Walton C. (2007). Impact of land-use change on dengue and malaria in northern Thailand. Ecohealth..

